# A Comparison between the Two Schools

**Published:** 1896-10

**Authors:** E. L. Close

**Affiliations:** Secretary


### A COMPARISON BETWEEN THE TWO SCHOOLS.

(Proceedings of the Brooklyn Hahnemannian Union.)

Before the formal opening of the meeting of the Brooklyn Hahnemannian Society, held April 25th, 1896, Dr. Baylies spoke of using Calcarea-bromata in a case of croup which indicated the same symptoms as Bromine and Calcarea, and Mercurius-bromata (30th) cured membranous croup in a Bromine constitution with mercurial symptoms.

Dr. Close felt a hesitancy about trying remedies not well proved, but Dr. Baylies said the elements were proved, and he saw the similarity of each. Dr. Close preferred looking for the single remedy, and thought the alternates might assail us, but Dr. Baylies said it was not a compound in the potency, but a unit.

The minutes of the last meeting were then read and approved, after which Dr. Anna Carman presented her paper on "A Comparison Between the Two Schools of Medicine," for, she said, although there are three schools, the eclectics have been included with the allopaths. There is a great distance between the homœopaths and the allopaths, which can only be lessened by drawing them to us. Patients yield to a treatment they abhor. Do the allopaths really believe what they practice? The greatest of them, after fifty years of such practice, acknowledge that they are working in the dark. In their eager seeking for new remedies, are they not seeking new light? How shall they find it? If *The Organon* held a higher place the average homœopath would be a centre of right influence. Argument does not win, but speedy restoration will.

Dr. Lutze feared the medical millennium would never come, but Dr. Baylies thought the majority would in time accept the truth.

Dr. Lutze referred to the reunion of his class every five years; that it was amusing to hear the various opinions from one *Alma Mater*—some high, some low, some alternates, and others openly using drugs to suppress pain.

Dr. Carman expressed surprise to find the strange way in which some so-called homœopaths treated their patients.

Dr. Close thought true practice depended upon something else than intellect, for that may turn to pathological problems; that it required a conscientious following of open vision of the truth.

Dr. Baylies added, above all, it requires conscientious study and interest in the good of the sick.

Dr. Close suggested cultivation of the gentler side of human nature, as Dr. Carman had referred in her paper to the cruelty of the old-school practice, which tends to make men hard.

Dr. Baylies said there was needed also industry and a devoted application in order to learn to analyze and compare the medicines in their healing relation. One must do it perseveringly and for a long time, with considerable dissatisfaction, before he reaps a reward of sufficient knowledge.

Dr. Close, referring again to cruelty, said: "And yet the old-school physician is very conscious of the pain, quick to alleviate by narcotics, which the homœopath refuses to do, because, seeing beyond, he waits. The difference is in the bent of the mind. One man is superficial, not philosophical; the other delves, and knows that the highest end is gained by putting aside palliatives."

Dr. Baylies thought the allopath who does not know any other way is more conscientious than the homœopath who knows better and does it not. At which Dr. Lutze and Dr. Close both said that they didn't know it, they are not taught it in the schools, and Dr. Lutze quoted one of the professors, who said: "In malaria you must overcome the tendency with Quinine, then cure the patient afterward;" and the same in the use of Morphine—help the pain first and then cure. But Dr. Lutze added that he never could cure until the drugs were eliminated from the system.

Dr. Carman related the case of a young lady who came to her with real physical pain. Not knowing just what to give her at that time, she put up for her Sac-lac., and told her to call the next week, which she did, much better, and continued improving.

Dr. Carman asked: "Shall I continue her coming and giving Sac-lac., or shall I prescribe on past symptoms?"

Dr. Lutze said to keep her under observation, and Dr. Baylies thought best not to prescribe a remedy till the symptoms returned.

Dr. Close said: "Dr. Carman has given real curative influence to that patient and earned every dollar. She has followed psychological laws and obtained a real result, derived from a real influence just as powerful as the remedies, the power of suggestion; which is another method of calling into operation the healing power of life. This may be brought about by suggestion, by remedy, or by sheer effort of one's own will. It is not the medicine that cures, it is the life-force."

Dr. Baylies asked : "Medicine does not cure? You believe in the benefit of homœopathic selection and application? Now, if the homœopathically selected remedy induces the curative life-force, does not the homœopathic medicine cure? If it excites the restorative action, does it not cure? We admit that mental agency inducing confidence, the healing process becomes active. But do we not often find that in spite of the confidence patients have in us this restorative action is not set up? Why?"

Dr. Close said : "Investigation has shown that the reason for such failure, as well as for the occasions where we have a well-selected remedy and fail, is found in the surroundings. Once admit the influence of the mind and we can't confine it. Remedy or suggestion may be used on a patient, and why does he not recover? Because of counter suggestions from friends."

Dr. Baylies said : "Yes, and you find yourself robbed of your most devoted patients in that way, even when you know your skill affords a most reasonable success. We become sensitive to it. That is a rock of adversity."

Dr. Lutze told of just such a case, a young woman on a farm, who looked healthy, but was nervous and easily annoyed. Obtaining no relief from well-selected remedies, he found all the trouble was caused by a brother who tormented her. She afterward went to a woman's hospital and recovered, he thinks not because of the treatment and operation, but because relieved from the evil influence. Referring to Dr. Carman's question, he added that Prof. Lilienthal said Sac-lac. never did any harm.

Dr. Baylies said that some give tablets, thinking people will respect them more, but that he continued powders.

Dr. Close told of a patient whose face clouded over the little powders. So, one day, he gave him a small vial of sugar tablets, and he brightened up, feeling that *now* he had something; and he really improved.

After some conversation on mental healing between Dr. Baylies and Dr. Close, the meeting adjourned, Dr. Fincke being nominated chairman for May.

E. L. Close, *Secretary.*

